# The Dynamics of Honesty: Modelling the Growth of Costly, Sexually-Selected Ornaments

**DOI:** 10.1371/journal.pone.0027174

**Published:** 2011-11-02

**Authors:** Sean A. Rands, Matthew R. Evans, Rufus A. Johnstone

**Affiliations:** 1 Centre for Ecology and Conservation, University of Exeter in Cornwall, Penryn, United Kingdom; 2 Department of Zoology, University of Cambridge, Cambridge, United Kingdom; 3 Centre for Behavioural Biology, School of Veterinary Science, University of Bristol, Bristol, United Kingdom; 4 School of Biological and Chemical Sciences, Queen Mary, University of London, London, United Kingdom; UC Santa Barbara, United States of America

## Abstract

The handicap principle suggests that individuals of superior quality can more easily bear the cost of developing extravagant ornaments. Consequently, ornament size should provide reliable information about quality or condition. Previous models have largely ignored the process of ornament growth, focusing only on final ornament size. We model ornament growth schedules for individuals of different qualities, where higher quality individuals experience lower costs of carrying energy reserves of a given size, but where all individuals pay a net cost of carrying ornaments of a given size. If the costs of ornament production ensure that final ornament size reliably signals quality, the information conveyed by the signal can change dramatically during growth. Higher quality individuals should delay growth until closer to breeding. Taking a snapshot of partially developed ornaments prior to breeding would show them to be larger in poorer quality individuals. The claim that costly ornaments honestly signal quality thus needs to be understood in a dynamic context, and may only hold during some phases of growth.

## Introduction

Many sexually selected traits and ornaments such as horns and tails require an extended period of growth before they reach a mature state [1]–[4]. Much research [5]–[7] has examined how carrying large ornaments could handicap [8] an individual (where only good quality individuals should be able to cope with large costs resulting from the ornament), but little research has examined how the costs of producing and bearing an ornament shape its growth trajectory. Sexually selected traits are frequently characterized by extreme variation seen within populations [5], and we would therefore expect similar variation in growth trajectories. Only a few models, however, have focused on the process of ornament development: Aparicio [9] uses a cost-benefit approach to examine how fluctuating asymmetry can develop, whilst Badyaev [10] considers the evolution of cost-reducing developmental mechanisms. However, neither of these consider how a trait should develop within individuals. Bonduriansky & Day [11] and Lindström *et al*. [12] explore the trade-off between body growth and ornament growth using deterministic dynamic models, and Kokko looks at the development of ornaments at consecutive mating seasons [13]. Lastly, Lindström *et al*. [14], to whom we return, employ stochastic dynamic programming to model sexual signal dynamics in the face of random variation in resource availability.

In this paper, we develop a stochastic dynamic program (SDP) that examines how ornaments can develop in an adult animal that breeds at a set point of its lifecycle, assuming that it is investing its resources in either metabolic processes or the growth of the ornament–consequently, we do not focus directly on allometric relationships [15], [16], but rather the investment strategy of an animal challenged by environmental variation (although the latter may give rise to allometric patterns of growth – see [17]). Stochastic dynamic programming [18]–[20] is the perfect technique to examine how a trait should develop when we are able to quantify the fitness of an individual possessing an ornament of a specific size at a defined moment in time, and here we use a variation on a standard forage-rest model [19]. We consider an animal that can choose between two activities (low-cost resting and high-cost foraging), which allows us to incorporate an ornament-dependent cost that impacts on the amount of resources that the animal can collect to fuel further ornament growth.

Our approach is most similar to that of Lindström *et al*. [14], who also use dynamic programming to model allocation of resources to ornamentation in the face of stochastic variation in resource availability. However, they consider expenditure *during* the breeding season on a flexible signalling trait that may fluctuate in its level of expression. By contrast, we focus on cumulative expenditure *prior to* the breeding season on the growth of an ornament such as a horn or tail, which (once growth is completed) will not subsequently fluctuate in size. We show that differences in individual ‘quality’ (taken here to be correlated with an individual's energetic expenditure) can lead to very different growth schedules within the population, with implications for the dynamics of honesty.

## Methods

### Overview of Model

In this model, we consider the growth of an ornament over one hundred consecutive periods prior to the point at which mate choice occurs. At the beginning of each of these periods, the animal is assumed to make a decision about whether it forages or rests during the period. If it rests, it loses energy. If it forages, it uses energy but also has a chance of finding food, such that its energy will, on average, increase if it forages. (This is similar to standard assumptions of forage-rest dynamic programs [18], [21]–[25], although we do not consider predation risk in this example). The amount of energy it can gain from the environment is assumed to be dependent upon the size of the ornament it carries: ornament size is negatively related to mean gain from the environment. As well as deciding whether to forage or rest, at the beginning of each period the animal also decides whether to allocate a quantity of its energy reserves to growing its ornament.

We assume that the animal's fitness (assumed here to be some measure of reproductive success) is positively related to the size of the ornament (regardless of the final energy reserves of the individual) at the end of the final period considered in the model, which means that we can assign a fitness value to all possible combinations of the animal's state (defined here as the combination of its energy reserves and ornament size) at this final moment. By making this assumption, we can then use stochastic dynamic programming [18]–[20] to assign fitness values to all possible combinations of ornament size and energetic reserves at the penultimate moment before mating. This is done by considering all possible behavioural decisions (choosing to forage or rest, and how much energy to allocate to ornament growth) for each possible state the animal could be in. The mean fitness for each decision can be calculated by considering what states the animal could achieve in the final period if it makes a particular decision in the penultimate period, where each of these final states has a defined fitness value. Therefore, for each possible state in the penultimate period, it is possible to identify the optimal behaviour, which yields the highest mean fitness for the individual (which is consequently the fitness recorded for that state during the penultimate period). Having calculated the optimal behaviours and their associated fitnesses for all possible states in the penultimate period, the process can be repeated for the period immediately before the penultimate one, and consequently repeated again and again, moving one decision period back in time at each step. In this way, the optimal behavioural policy can be determined for the individual, which defines its optimal behaviour at any moment in time if its current energy reserves and ornament size are known. The techniques described above are standard for stochastic dynamic programming models. For further explanations of some of the standard assumptions and techniques used, see [18]–[20], [23], [26].

### Details of Dynamic Program

We examined the growth of ornaments over a period of *T* = 100 consecutive periods. During each period (denoted by *t*), the state of an individual has two components: *x*, its energetic reserves, and *s*, the size of its ornament. At the beginning of each period, the animal makes two decisions about its actions between *t* and *t*+1: *u*, its behaviour during the period (to either *forage* or *rest*), and *i*, the investment in ornament-building done during the following period (for simplicity, we assume the animal can decide to increase its ornament by 0 or 1 units). To introduce some stochasticity to avoid grid effect artefacts [18], after choosing to invest in the ornament, the investment is successfully increased with likelihood *d* (the alternative being that investment is unsuccessful, with no increase in the ornament occurring despite incurring expenditure). Considering a simple discrete choice of investing 0 or 1 units into an ornament may well introduce some constraints upon the form of growth of the ornament within the model, but should give us a rough understanding of investment decisions over time. Further exploration could involve identifying the optimal probability of investment during a period (instead of a simple choice to invest or not), but is unlikely to make a difference to the qualitative results presented here.

The energetic costs incurred by the animal are dependent upon its behaviour (characterized by a base level of expenditure *K_u_*), where foraging is assumed to be more expensive than resting. Investment *i* in an ornament also incurs energetic expenditure. Finally, energy use can also depend upon the size of the energy reserves and ornament, for instance through mass-dependence [27]: we modelled these expenditures using scalars (*k_x_* and *k_s_*) and power constants (*p* and *q*) to describe potentially non-linear increases in expenditure with reserves (*k_x_* and *p*) and ornament size (*k_s_* and *q*). Because the mean expenditure (calculated as *K_m_* = *K_u_* + *i* + *k_x_ x^p^* + *k_s_ s^q^*) from these variables could take a non-integer value (energetic reserves themselves are in integer units), we calculated κ(*c*; *x*, *s*, *u*, *i*), the probability of expending *c* units of energy (where *c* = 0, 1, 2, 3, or 4 energy units) when in state (*x*, *s*) and conducting actions (*u*, *i*), as a discretized normal distribution with a mean value of *K_m_* and standard deviation of *v_c_*.

If the animal chooses to forage and is unhampered by any kind of ornament (*s* = 0), the base level of energy found in the environment is *b_g_*. However, carrying an ornament could lead to a reduction in foraging efficiency dependent upon ornament size (modelled using a scalar *r_s_*), and mean energetic gain is therefore calculated as *G_m_* = *b_g_*−*r_s_ s*. Again, because mean intake can take a non-integer value, and also because finding the food may depend upon the environment, we calculated the probability of gaining *g* = 0, 1, 2, 3, or 4 energy units, γ(*g*; *s*), based upon a discretized normal distribution with mean *G_m_* and standard deviation *v_g_*.

The fitness of the animal is determined by the reproductive success it achieves when it reaches the end of the period modelled (assuming it survives to this period): at *T*, we assume that reproductive success depends upon the size of its ornament, and is defined by the reward function
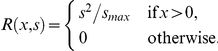



This is taken to be an accelerating function of ornament size. Because the results of this model are potentially highly dependent upon this reward function, we also investigated linear and decelerating increases in fitness, and obtained results that were qualitatively similar to those given below. Further consideration of the form of the reward function may be useful for exploring how variation in mating success correlates with individual condition and quality [28], but the broad approach we use here gives a broad qualitative indication of how ornaments may develop within a population.

The function *V*(*x, s, t*) is the fitness at time *t* for an animal in state (*x*, *s*). The dynamic programming equations were set so that at *t* = *T*, *V*(*x, s, T*)  = *R*(*x*, *s*), whereas when *t*<*T*,



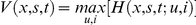
,

where, if *x* = 0, 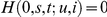; otherwise


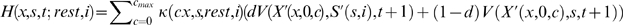
, and



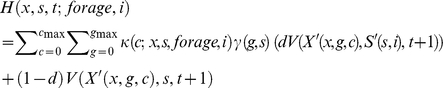



using chop functions *X′*(*x*, *g*, *c*)  =  min(max(*x*+*g*−*c*, 0), *x*_max_) and *S*′(*s*, *i*)  =  min(*s*+*i*, *s*_max_): these chop functions limit state variable changes to within maximum and minimum values [20]. *V*(*x, s, t*) is calculated by backwards induction, as standard in SDP models [19], [20]. The values of *u* and *i* that maximize each *V*(*x, s, t*) are recorded. These are then used in forward simulations, which assume that at *t* = 0, ornament size *s* = 0, and reserves *x* are low: writing *in*(*x*, *s*) for the proportion of the population in state (*x*, *s*) at *t* = 0, we assumed that *in*(1, 0)  =  *in*(5, 0)  = 0.1, *in*(2, 0)  =  *in*(4, 0)  = 0.2, *in*(3, 0)  = 0.4, and *in*(*x*, *s*)  = 0 for all other state pairs.

The model was coded in C++, using long double floating point precision to minimize computational artefacts [29].

### Sensitivity Analyses

We conducted sensitivity analyses to investigate the relative effect of each of the variables used in the model. Using a framework with *s_max_*  = 50, and *x_max_*  = 100, we used a Mersenne twister algorithm to randomly generate 50 independent parameter sets, where the values of the parameters were taken from uniform distributions with the following ranges: *b_g_* ∈ [0.5, 2.5], *d* ∈ (0, 0.5], *K_f_* ∈ [0.5, 2.0], *k_s_* ∈ [0.0001, 0.5], *k_x_* ∈ [0.0001, 0.5], *p* ∈ [1.0, 2.0], *q* ∈ [1.0, 2.0], *r_s_* ∈ [0.0001, 0.1], *ν_c_* ∈ [0.5, 1.0], *ν_g_* ∈ [0.5, 1.0]. The randomly generated value of *K_r_* was dependent upon the value of *K_f_* generated, and came from the range [0.1, 0.5× *K_f_*]. For each of the 50 parameter sets, we calculated policies and the resulting population distributions when each of the parameters was changed systematically (holding all the other parameters at the initial randomly generated value): for a given focal parameter π we calculated these for 0.1×π, 0.2×π, … , 2.0×π.

To assess how changing the size of each parameter affected the final ornament size, for each of the fifty parameter sets we calculated the mean ornament size within a population in response to the size of the focal parameter, and then used *R* 2.2.0 [30] to fit a least-squares regression line (considering final ornament size in response to a standardised size of the focal parameter, such that the standardised parameter was a value from the series 0.1, 0.2, …, 2.0). The predicted slope of the line was recorded, ultimately giving us 50 regression coefficients for each of the variables considered, which were then summarised to give a median value and associated ranges. It should be noted here that although final ornament size was very unlikely to follow a straight line, we felt that fitting a straight line was sufficient for exploring how changes in the value of a parameter could affect the final size of the ornament, as most cases (as described below) showed a gradual increase or decrease in ornament size, and we required a simple statistic to compare the spread of data between the variables.

For each of the 50 replicates, we also identified the period at which at least 50% of the population had started growing an ornament (so that *s* ≥1). We quantified how this changed using the regression coefficient technique described above.

## Results

**Figure 1:**
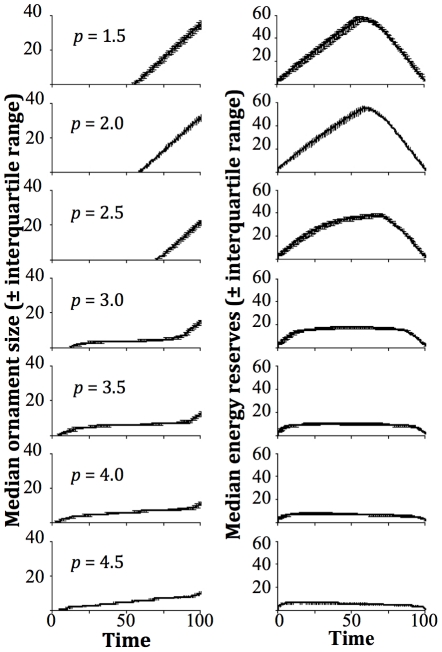
Changes in ornament growth and reserve size with increasing costs of carrying large reserves. In successive panels, the relative energetic cost of carrying large energy reserves is increased by increasing the power term *p*. *b_g_* = 2.0, *d* = 0.8, *K_f_* = 1.0, *K_r_* = 0.5, *k_s_* = 0.0001, *k_x_* = 0.0001, *q* = 2.0, *r_s_* = 0.1, *s_max_* = 50, *T* = 100, *ν_c_* = 0.5, *ν_g_* = 0.5, *x_max_* = 100.

The physiological parameters included in the model had broadly similar effects, only differing qualitatively in their ‘direction’. Figure 1 illustrates the typical sequence of changes in response to systematically changing a parameter, while table 1 outlines the direction of changes (up or down the sequence of graphs in the figure) induced by increasing each different parameter. For illustrative purposes (figure 1), we have shown how optimal ornament growth changes as the energetic expenditure of carrying energy reserves is increased (holding the other physiological costs constant), but the other parameters listed in Table 1 all induce similar effects as they are increased or decreased.

**Table 1:** Changes in ornament growth pattern obtained by increasing the parameter values.

parameter	description	change with increase in parameter
*b_g_*	basal energetic gain	↑
*d*	probability of a correct investment in ornament	↑
*K_f_*	basal energetic cost of foraging	↓
*K_r_*	basal energetic cost of resting	↓
*k_s_*	ornament cost scalar	↓
*k_x_*	reserve cost scalar	↓
*p*	reserve cost power term	↓
*q*	ornament cost power term	↓
*r_s_*	scalar for energy intake reduction due to ornament	↓
*v_c_*	cost standard deviation	↓
*v_g_*	gain standard deviation	↑

The arrows refer to the direction of change sketched in figure 1, where ‘↓’ indicates an increase in the parameter gave a change similar to moving from top-to-bottom in figure 1, and ‘↑’ indicates a reversed (bottom-to-top) change.

In this illustrative example, where the relative cost of carrying energy reserves is small, signal growth is initiated late in the developmental period, and the signal is grown at a maximum rate up to the end of the developmental period, giving near-linear growth (seen in figure 1 where *p* = 1.5). To begin with, as the cost of carrying reserves increases, the time at which the signal is initiated moves later in the growth period (*p* = 2.5), though initiated growth is still linear. Initiation occurs later because the optimal size of the final signal is reduced, but the animal is still able to reach this size if it leaves investing until as late as possible, and then grows the signal at a maximum rate.

As the cost of carrying energy reserves increases further, however, there comes a point at which the cost of producing and supporting the maximum ornament is too much, and so the final size of ornament produced is not the largest possible. From this point, the models frequently show that the animal doesn't invest in growth in a linear manner. Instead, early investment is put into producing an initially small ornament, and then further investment is put into the ornament just before the final period (*p* = 3.5). This initial investment in a small ornament is likely to occur as a buffer against a variable environment, where the investment and less-costly carrying of a small initial ornament ensures that the animal can then build the costlier part of the ornament prior to its need, and gain the highest reward possible. However, as the cost of carrying energy reserves increases further, the price of building and maintaining even a mediocre ornament is such that any investment starts later and later in the growth period (*p* = 4.5).

Figure 1 also demonstrates that the animal will tend to show a peak in its energy reserves in the middle of the developmental period, and these are then reduced as it begins to invest heavily in the ornament. If costs are high, the maximum level of reserves that the animal can maintain and carry is reduced, which in turn affects the amount that it is able to invest in its ornament.

**Figure 2:**
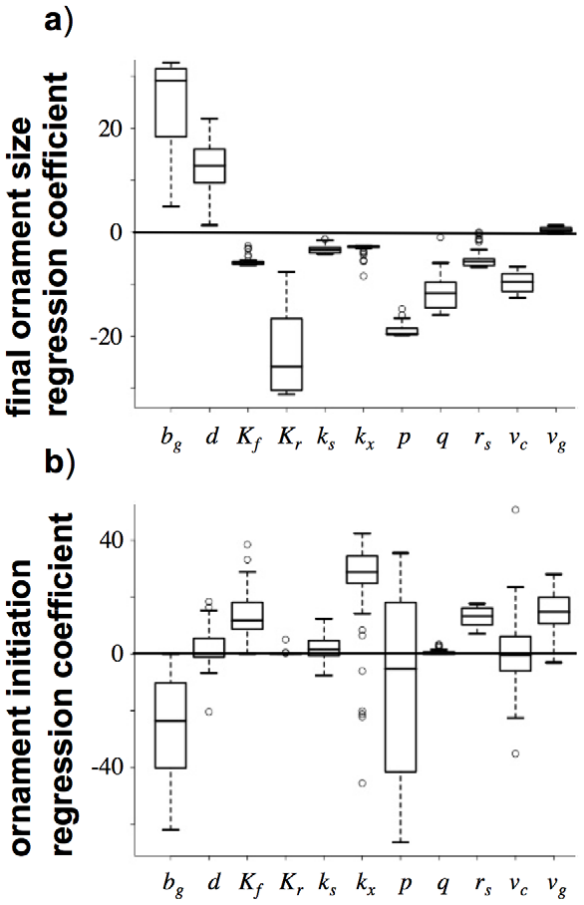
Results of sensitivity analyses. Boxplots describing the median size (with corresponding interquartile and 95% ranges) of the regression coefficients for **a**) final ornament size and **b**) period at which at least half of the population have initiated ornament growth, obtained for the eleven variables described in the model (for full meanings of the parameters, see Table 1), resulting from the exploration of parameter space described in the sensitivity analyses. A large positive median value of the coefficient shows that final ornament size/start period increased greatly as the size of the variable being investigated was increased (and a large negative value showed that final ornament size/start period decreased as the variable was increased). A large amount of variation around the median suggests that the amount that the ornament or period can vary is sensitive to the parameterisation of the system.

Figure 2 shows that the basal rate of energy gain *b_g_* has a large effect upon both the final size of the ornament (Figure 2*a*), and when ornament growth is initiated (Figure 2*b*). This is to be expected as the animal is trading off ornament growth with the risk of starvation, and so a reduction in the baseline amount of food in the environment should have a large effect upon ornament scheduling. The final ornament size (Figure 2*a*) is also greatly affected by the cost of resting *K_r_*, where if resting gives relatively little reduction in energy expenditure compared to foraging, carrying an ornament becomes relatively more costly and so growth is delayed. The other parameters had varying degrees of effect upon final ornament size with respect to how the variable of interest was altered, but show little variation with different parameter sets, suggesting that differing costs of carrying an ornament or energy reserves have little overall effect upon growth scheduling. The period at which ornament growth is initiated also remains relatively unaffected by parameter sets with two exceptions: the basal rate of energy gain (as described above), and the reserve cost power term *p*. We took an initial random value of *p* between 1 and 2, and then calculated a range of ×0.1 to ×2 around this, meaning that *p* could potentially range between 0.1 and 4, and so we were considering a cost of energy reserves that could deceleratingly or acceleratingly increase with reserve size. This means that if the cost of carrying reserves increases in a potentially non-linear manner, this can have a large effect upon the growth schedule seen, and the shape of this function should therefore be considered carefully if it is being used in a predictive model. These sensitivity analyses show that these patterns are extremely robust to changes in the various parameters built into the model (table 1), as well as changes in the number of periods, states, and probabilities considered. The trends apparent in table 1 are as would be expected, where increasing variables that could lead to energetic costs increasing (such as through increasing the cost of carrying an ornament) saw a move away from building an ornament early to late investment.

## Discussion

Careful attention needs to be paid to how the growth sexual ornaments are scheduled by individuals. The model developed here suggests that the pattern of ornament development can be highly variable. The fact that investment and maintenance should be reduced as an ornament becomes more energetically costly isn't surprising, but the fact that an individual may carry a partly-developed ornament for a long period of time before allowing it develop fully is important. For example, if an animal develops a costly ornament well in advance of the breeding season, it could be argued that carrying this costly ornament for a long time is a manifestation of the handicap principle [8], as carrying a costly ornament for a long time would arguably only be possible in a good quality individual. This would in turn assume that mates were able to assess the quality of individuals based upon their monitoring the appearance and growth of ornaments early in the season, prior to the mating period. Our model doesn't make this assumption however: we assume that no mate assessment occurs before the period of mating (which could be the case for a lekking species where individuals are solitary prior to the breeding season), and therefore the early development of ornaments simply ensures that the individual can possess the best possible ornament when it begins to breed.

**Figure 3:**
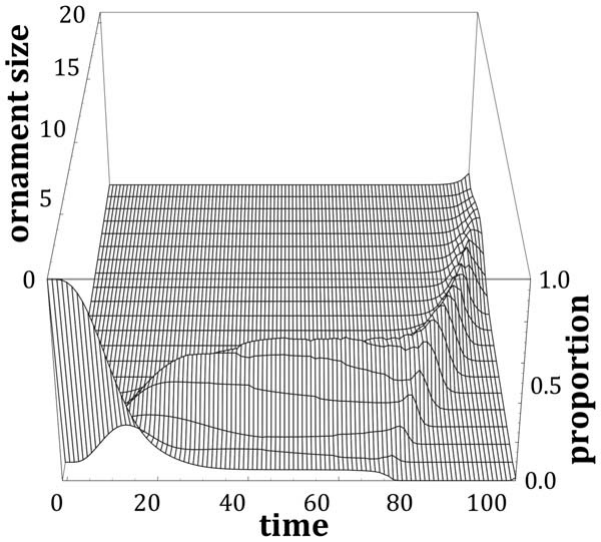
Ornament development over time in a population of individuals with differing ‘qualities’. Although ornaments increase in size, there is considerable variation within the population. Here, the population consists of individuals of 21 quality types, defined by the individual's energetic expenditures when carrying a given amount of energetic reserves. The policies are derived for individuals with *p* = 2.0, 2.1, ..., 4.0 (*s_max_* = 20, other parameters as figure 1). The initial proportion of each type follows a binomial distribution, where the likelihood of an individual following a policy with reserve cost power *p* is _20_C_((*p*–2)/10)_⋅(0.5)^20^.

**Figure 4:**
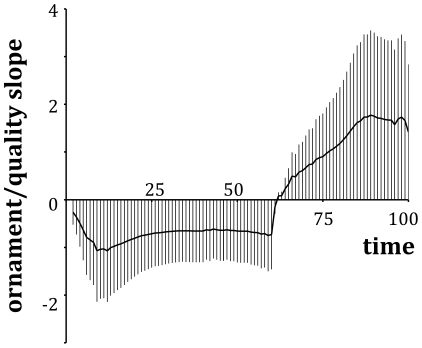
The relationship between quality and ornament size changes over time. Early in ornament development, individuals with large ornaments tend to be poor quality (shown by a negative value of ‘ornament/quality slope’), but later on, quality is positively related to ornament size. In this example, we define the quality of an individual as *Q* = −10 *p*. Using the quality proportions calculated for figure 2, we regressed (using least-squares regression) the mean value of *Q* for a given ornament size in a period against size. The ‘ornament/quality slope’ is the slope (± s.d.) of the calculated best-fit line.

This framework can also be used to consider what should be happening within populations of individuals that differ in their ability to respond to environmental stochasticity. This difference in what we could describe as quality may lead to a difference in investment between individuals, as their optimal policies will be based upon different parameter sets (where we can use our findings from the sensitivity analyses to identify where variation will be important). At any point in time, there will be a large amount of variation in ornament size within the population (Figure 3). However, as we have shown above, it may not be the case that the individuals with large ornaments earlier on in the developmental period are the higher quality individuals. Figure 4 illustrates this by considering how the distribution of ornaments over time shown in Figure 3 relates to the qualities of the individuals within the population. In Figure 4, there is a negative relationship between quality and ornament size for the first two thirds of the developmental period, and it is only after this point where high quality individuals will tend to have larger ornaments. Therefore, if we are assessing the quality of individuals prior to breeding, we must be careful to ensure that, as well as the inferences we make about quality from measurements, we also take into account the fact that poor quality individuals could have larger ornaments than high quality individuals for a large proportion of their developmental schedule. It should also be acknowledged that for some, flexible, types of display, the signals produced by individuals may change dynamically over the course of the breeding season, and individuals starting with high quality signals may show a reduction in the quality of their signals over time [14].

It is difficult to assess how important this effect is, for little data exists that examines how ornaments develop in an individual through the course of a breeding season or lifecycle: although measurements of the growth of ornaments can be done routinely, (*e.g.* [31]–[35]), measurements so far taken to compare the size of ornaments between individuals have only been made at a single point of the season (*e.g.* [36]–[38]). There is therefore much to be gained by examining the ornament growth schedule of known individuals within populations, relative to their seasonal mating success, where modelling work using the framework described above would give new insights into individual variation in signal production. Furthermore, the modelling framework could be extended to consider life histories [39] where traits develop multiple times, or where potential mates can track and assess the quality of an individual throughout the life of the signaller, rather than at a set moment in time.

We also need to assess how ornament growth relates to sexual selection processes where mate choice and competition between individuals is also important. The model presented here does not make any inferences about how selection could shape the reward offered to individuals with differing ornaments, and would need to be extended in order to consider how ornament development is tied in with how sexual selection shapes mate preferences within populations. At the moment, the fitness return to an individual that has an ornament of a given size in the period when mate choice occurs is represented by a fixed function, but it is highly likely that the fitness return of a given ornament needs to reflect what else is available in the population. This means that ideally we should take the social context of the signal into account (which could be done here by extending the dynamic program presented here into a dynamic game between population members, *e.g.* [21], [22], [40]), where the state-dependent rewards associated with mating will depend upon the frequency of differently sized ornaments in the population. It is consequently difficult to predict the exact effects that a changing population distribution of ornaments would have on the growth schedules of individuals of different qualities. Furthermore, we do not consider whether the animal is constrained in its ability to judge or perform the optimal behaviour [41], or whether it instead has to use a rule-of-thumb that approximates this optimal policy [42]–[44]. Taking these considerations into account, we strongly suggest that the framework we describe here should be extended to consider a dynamic game between individuals [14], [18], [21], [22], [40], and we are confident that this technique could open many new avenues of research into an unconsidered side of sexual selection.
